# Optimizing clinical nutrition practices: impact of a formulary prescription-based nutrition therapy protocol in surgical patients

**DOI:** 10.3389/fnut.2025.1733325

**Published:** 2026-01-21

**Authors:** Mengying Xu, Jiaojiao Mao

**Affiliations:** Department of Pharmacy, The Fourth Affiliated Hospital of Soochow University, Suzhou Dushu Lake Hospital, Medical Center of Soochow University, Suzhou, Jiangsu, China

**Keywords:** parenteral nutrition, formulary prescription, nutrition therapy, surgical patients, gastrointestinal cancer

## Abstract

**Background:**

Parenteral nutrition (PN) is a crucial clinical therapy, particularly in surgical patients. However, due to the complex composition of PN formulations, the inappropriate use of PN remains widespread in clinical practice to date. This study established a formulary prescription-based nutrition therapy protocol and assessed the effectiveness of its implementation.

**Method:**

The formulary prescription-based nutrition therapy program included the development of formulary prescriptions for PN and the establishment of a perioperative nutrition medication pathway based on the formulary prescriptions. To evaluate the effect of this protocol in clinical practice, a before-and-after cohort analysis was performed to compare PN administration modalities hospital-wide and nutrition therapy strategies in targeted surgical (general surgery and thoracic surgery) departments. Patients admitted in hospital with gastrointestinal (GI) cancers and receiving abdominal surgery during the study period were further retrospectively analyzed. Nutrition status of patients was evaluated using the levels of postoperative serum albumin and prealbumin. Recovery time and surgical or nutrition-related complications and were also collated as the clinical outcomes.

**Results:**

The proportion of all-in-one (AIO) admixture orders increased from 47.2 to 85.2%, while multi-bottle systems (MBSs) decreased from 52.8 to 14.8% (*p* < 0.01). In targeted surgical departments, the utilization rate of personalized total nutrient admixture (TNA) increased from 54.5 to 79.3% (*p* < 0.01), and a concurrent shift in the approach of nutrition therapy from total parenteral nutrition (TPN) to supplemental parenteral nutrition (SPN) and total enteral nutrition (TEN). Significant benefits across the spectrum of postoperative outcomes were observed in patients with GI cancers. The average postoperative prealbumin levels were elevated on both postoperative day 3 (POD 3) and postoperative day 5 (POD 5) in the after cohort compared to the before cohort (114.40 ± 41.95 vs. 130.09 ± 38.55 mg/L on POD 3 and 131.45 ± 36.88 vs. 148.02 ± 33.69 mg/L on POD 5). In addition, the after cohort showed shorter duration of postoperative PN therapy (8.2 ± 3.3 vs. 6.3 ± 2.6 days) and shorter length of postoperative hospital stay (14.9 ± 5.1 vs. 12.2 ± 4.2 days). However, there was no significant difference in the incidences of postoperative complications between the two cohorts.

**Conclusion:**

The formulary prescription-based nutrition therapy protocol can significantly enhance the rational use of PN drugs hospital-wide, optimize clinical nutrition strategies in the surgical patients, and improve short-term clinical outcomes in the patients undergoing GI cancer surgery.

## Graphical abstract

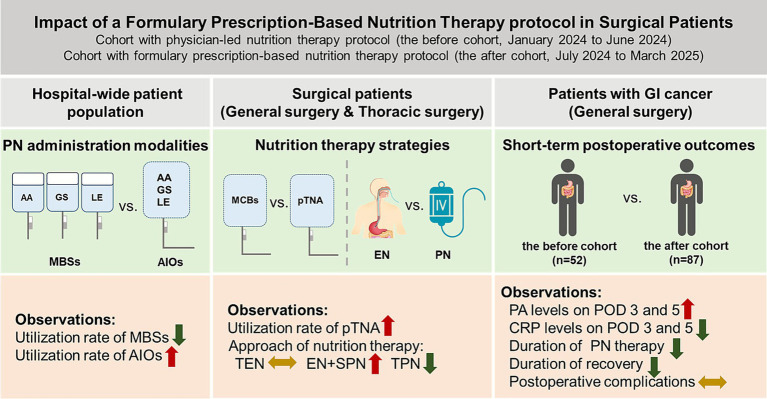

## 1 Introduction

Globally, 30–45% of hospitalized adults were malnourished on admission (1). The estimated prevalence of malnutrition in patients after major surgery ranges from 20 to 70% (2). Due to the special surgical site and worse nutrition status before operation, the recovery time is longer in patients with gastrointestinal (GI) cancers compared to patients receiving other surgeries (3). It is well documented that the catabolic response to surgery causes the depletion of essential nutrients, resulting in an increased risk of postoperative complications, particularly infectious complications (4). Therefore, timely and rational nutrition therapy is essential for maintaining optimal cell and organ function, promoting wound repair, and decreasing infectious complications after surgery.

Despite identified nutritional risk or malnutrition, merely half of affected hospitalized patients in China received nutrition therapy (5). Notably, 68.6–91.5% of these interventions utilized parenteral nutrition (PN), which significantly deviated from current clinical guidelines recommending enteral nutrition (EN) as the preferred approach (6, 7). The misuse of PN primarily manifests in the following scenarios: (1) improper indications, (2) prolonged treatment duration beyond clinical necessity, and (3) suboptimal administration modalities, particularly the separate or sequential infusion of individual amino acid and/or lipid emulsion bottles rather than clinically recommended all-in-one (AIO) admixtures (8). In clinics, perioperative PN prescriptions are usually prescribed by surgeons. Our previous investigations (data not shown) showed that, due to workload and specialty limitation, the PN prescriptions issued by surgeons were often unsatisfactory. Multiple-bottle infusion and lack of individualization were common problems of PN. Sub-optimal PN prescriptions may not only affect the efficacy of the PN, but also affect the safety and clinical outcomes of patients.

As we all known, A formulary prescription refers to a standardized treatment protocol jointly developed by clinicians and pharmacists based on clinical needs, and subsequently approved by the pharmacy administration committee and hospital leadership as an institutional routine prescription. Formulary prescriptions are most prevalent in Traditional Chinese Medicine (TCM) due to the fixed compositions with scientifically validated herb ratios and demonstrated efficacy (9). These standardized prescriptions not only enhance prescribing efficiency, but also streamline the dispensing process by eliminating the labor-intensive steps of manual herb weighing and decoction preparation.

For PN, the rationale for implementing formulary prescriptions is twofold: (1) the complex prescription requirements involving precise calculations of energy needs, protein supply, non-protein energy (NPE), glucose-to-lipid ratios, osmolarity, and compatibility considerations and (2) variability in clinical expertise, where non-specialist physicians across departments independently formulate nutrition regimens without standardized training (10, 11). In view of this, clinical pharmacists with expertise in parenteral and enteral nutrition have developed a formulary prescription-based nutrition therapy protocol in our institution. Specifically, we integrated tiered nutritional formulary (graded by fluid, energy, and protein) in accordance with the specific demands of various clinical units into the physicians’ Hospital Information System (HIS). We enabled clinicians to select the most appropriate nutritional prescription based on the patient’s clinical condition. This initiative has successfully promoted the implementation of evidence-based pathways for perioperative nutrition therapy across surgical departments, significantly improving clinical practice quality and medication safety while serving as a replicable model for institutional PN management.

A retrospective study was conducted to evaluate the achievements of the formulary prescription-based nutrition therapy protocol. Firstly, as rational medication use is the paramount responsibility of pharmacists, we evaluated the rate of inappropriate PN administration across the hospital-wide patient population. Secondly, nutrition therapy requires frequent adjustment based on the patients’ evolving clinical conditions in surgical patients, we assessed the appropriateness of nutrition therapy strategies in the general surgery and thoracic surgery departments. Lastly, among patients with GI cancers, we examined the impact of the aforementioned interventions on short-term postoperative outcomes.

## 2 Methods

### 2.1 Development and optimization of formulary prescriptions for PN

An evaluation standard of perioperative PN was established by pharmacists based on the guidelines of the European Society for Parenteral and Enteral Nutrition (ESPEN) (12), the American Society for Parenteral and Enteral Nutrition (ASPEN) (13) and the Chinese Medical Association for Parenteral and Enteral Nutrition (CSPEN) (14). The formulary prescriptions for PN were initially piloted in the departments of general surgery and thoracic surgery, and subsequently expanded to departments of oncology, radiation oncology, geriatrics and intensive care unit. The formulary prescriptions for PN are primarily indicated for clinically stable patients. To accommodate interpatient variability in energy, protein, and fluid requirements, a series of supplemental PN (SPN) and total PN (TPN) formulations were developed with graded levels of these components. Specifically, the following customizable ranges were available: a fluid volume of 700–1,500 mL, a caloric content of 700–1800 kcal and a protein content of 20–80 g. Access-adapted formulations were created based on venous availability: peripheral PN (osmolarity <900 mOsm/L) for patients without central venous catheters and central PN (osmolarity≥900 mOsm/L) for those with central access (15). Additionally, specialized formulations were designed for hepatic dysfunction (branched-chain AA-enriched or SMOF-based regimens) and lipid emulsion allergies (lipid-free regimens). All nutritional parameters in these formulary prescriptions including the calorie-to-nitrogen ratio, glucose-to-lipid ratio, and electrolyte concentrations were strictly aligned with evidence-based guideline recommendations. The formulary prescription templates were embedded into HIS, with detailed annotations for each prescription including (1) nutritional parameters (energy, protein, fluid volume, and osmolarity), (2) administration route, (3) immune-nutrition components (e.g., alanyl-glutamine or fish oil-based lipid emulsions), and (4) special population indications (e.g., hepatic dysfunction). This system enabled clinicians to select patient-specific PN regimens based on pathophysiological status (e.g., stress hypermetabolism and severe infection) and dynamically adjust electrolyte compositions according to real-time laboratory results.

During the clinical implementation of formulary prescriptions, clinical pharmacists conducted timely maintenance and optimization of prescription templates. Key triggers for updates included changes in hospital nutrition product suppliers or specifications, required adjustments identified through pharmacovigilance. For instance, patients undergoing anti-neoplastic therapies (chemotherapy/targeted therapy/immunotherapy) often experienced compromised vascular integrity, resulting in a lower tolerance threshold for the osmolarity of PN administered via peripheral veins. It is therefore necessary to revise formulary prescriptions to accommodate peripheral venous administration in these individuals.

### 2.2 Establishment of a perioperative nutrition medication pathway based on the formulary prescriptions

**Figure 1:**
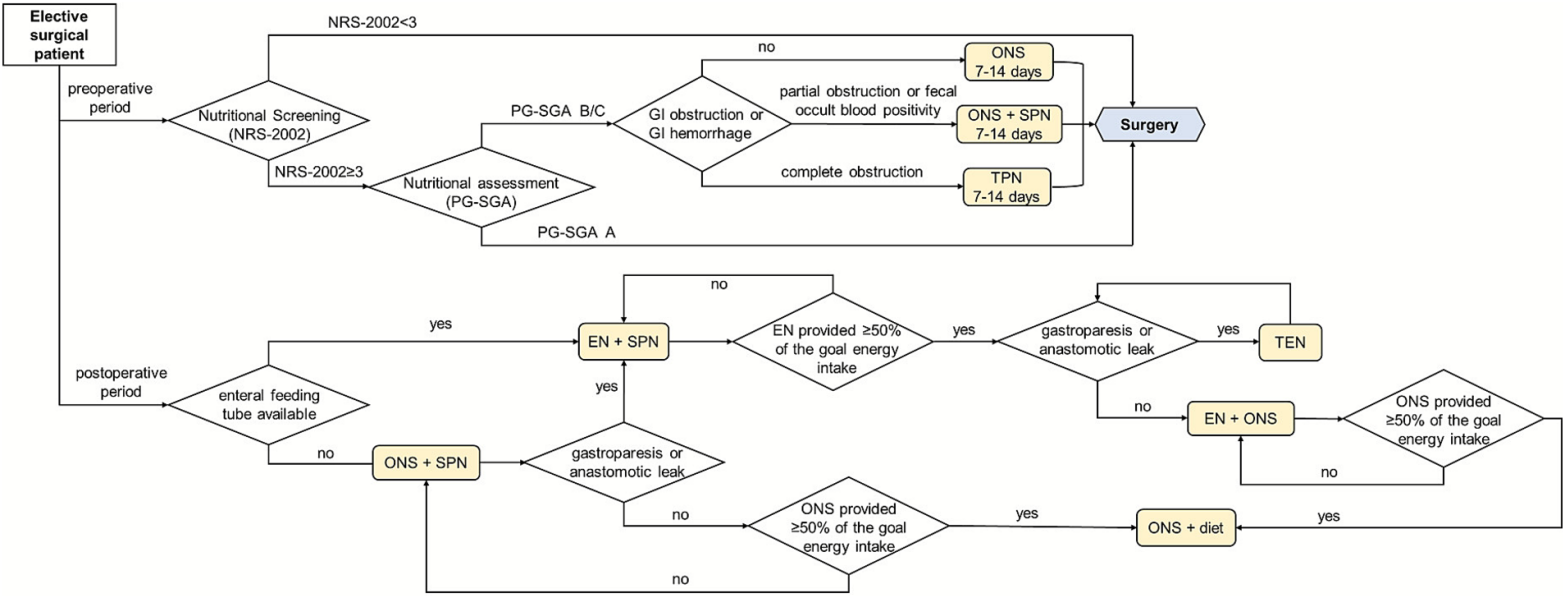
The pathway of perioperative nutrition therapy.

For patients with preoperative nutritional risk, early nutrition therapy significantly reduces postoperative complications and shortens length of hospital stay (16). Although early postoperative oral diet, oral nutritional supplements (ONS) and EN have achieved broad consensus (12, 17), their implementation is often delayed or reduced due to clinical factors including disease severity, GI tolerance of patients and physicians’ knowledge level of nutrition therapy. On the other hand, the ESPEN guidelines recommend that surgeons consider initiating SPN if the energy requirements (<50% of energy requirement) of the patient have not been met by total enteral nutrition (TEN) for more than 7 days (12). In other words, to supplement insufficient EN, SPN is a strategy that can increase energy delivery more closely to the estimated energy requirements. Therefore, clinical pharmacists have established a perioperative nutrition medication pathway based on the formulary prescriptions of PN by integrating clinical guidelines, consensus statements, practical experience, and surgical-specific considerations (Figure 1). It should be noted that PN prescriptions involved in the pathway were preferably selected from the formulary prescriptions.

### 2.3 Study design

A retrospective cohort study was conducted to assess the impact of the aforementioned work on nutrition medication use. The study was conducted in accordance with the Declaration of Helsinki and approved by institutional ethics committee of the Fourth Affiliated Hospital of Soochow University. We analyzed all PN orders for hospitalized patients during two distinct periods: the before cohort (January 2024 to June 2024) prior to establishing the formulary prescription-based nutrition therapy protocol, and the after cohort (July 2024 to March 2025). Additionally, a further retrospective analysis was conducted for patients with GI cancers admitted to the department of general surgery between January 2024 and March 2025.

### 2.4 Source of data

In the hospital-wide analysis of rational nutrition therapy utilization, we quantified utilization patterns across three PN administration modalities: (1) multiple bottle systems (MBSs), (2) commercially available multi-chamber bags (MCBs), and (3) personalized total nutrient admixture (pTNA), with particular focus on the change of nutrition therapy approaches (TEN, EN + SPN, or TPN) in the departments of general surgery and thoracic surgery. Data were collected through retrieving medication orders from the HIS and dispensing records from the Pharmacy Intravenous Admixture Service (PIVAS) system.

In the analysis of the impact of the formulary prescription-based nutrition therapy on short-term outcomes in perioperative patients with GI cancers, the clinical information of the patients and the PN related information were also retrieved from HIS. Patient characteristics included age, gender, BMI, NRS-2002 score, tumor type, hematological examination, etc. A total of 52 patients were initially enrolled in the before cohort and 87 patients were initially enrolled in the after cohort. The basic clinical information of the patients was shown in Table 1. There were no significant differences between the two cohorts (*p* > 0.05). The clinical outcome measures included (1) a component of postoperative nutritional indicators: albumin and prealbumin levels; (2) postoperative recovery indices: anal exhaust time, duration of abdominal drainage, length of postoperative hospital stay and length of total hospital stay; (3) duration of postoperative PN therapy and (4) postoperative complications: nutrition-related complications, infections, gastroparesis, intestinal obstruction and anastomotic leak.

**Table 1:** Baseline demographic and clinical characteristics^a^.

Characteristic	The before cohort (January to June 2024, *n* = 52)	The after cohort (July 2024 to March 2025, *n* = 87)	*p*-value
Gender, No. (%)			0.804
Male	31(59.6)	50 (57.5)	
Female	21(40.4)	37 (42.5)	
Age, y	69.4 (6.8)	70.3 (7.9)	0.581
BMI	23.2 (3.8)	22.9 (3.2)	0.619
NRS-2002 score, No. (%)^b^			0.979
3	27 (51.9)	44 (50.6)	
4	20 (38.5)	35 (40.2)	
≥5	5 (9.6)	8 (9.2)	
Diagnosis, No. (%)			0.734
Gastric cancer	12 (23.1)	25 (28.7)	
Colorectal cancer	35 (67.3)	53 (60.9)	
Others^c^	5 (9.6)	9 (10.3)	
Nutritional indicators			
Albumin, g/L	39.62 (4.32)	38.84 (4.43)	0.322
Prealbumin, mg/L	178.31 (56.88)	169.69 (57.37)	0.398
C-reactive protein, mg/L	8.44 (5.28)	9.21 (5.22)	0.617
Duration of surgery, min	191.1 (71.3)	196.3 (68.6)	0.681

^a^ Data were presented as mean (SD) unless otherwise indicated. ^b^ Scores on NRS-2002 range from 0 to 7, with a score of 3 or more identifying patients at nutritional risk. Higher scores indicate an increased risk. ^c^ gastric cardia cancer, cecal cancer or duodenal cancer.

### 2.5 Statistical analyses

The GraphPad Prism software (version 9.0, GraphPad Software, La Jolla California, USA) was used for statistical analyses. Continuous variables are expressed as mean ± SEM and categorical variables are expressed as counts and percentages. Students’ *t*-tests were performed to analyze the continuous data of two groups. Chi-squared (χ^2^) and Fisher’s exact tests were used to evaluate the differences between groups of categorical variables. For all tests, a two-sided *p* value <0.05 was considered statistically significant.

## 3 Results

### 3.1 Formulary prescription-based nutrition therapy improved the rationality of PN

**Figure 2:**
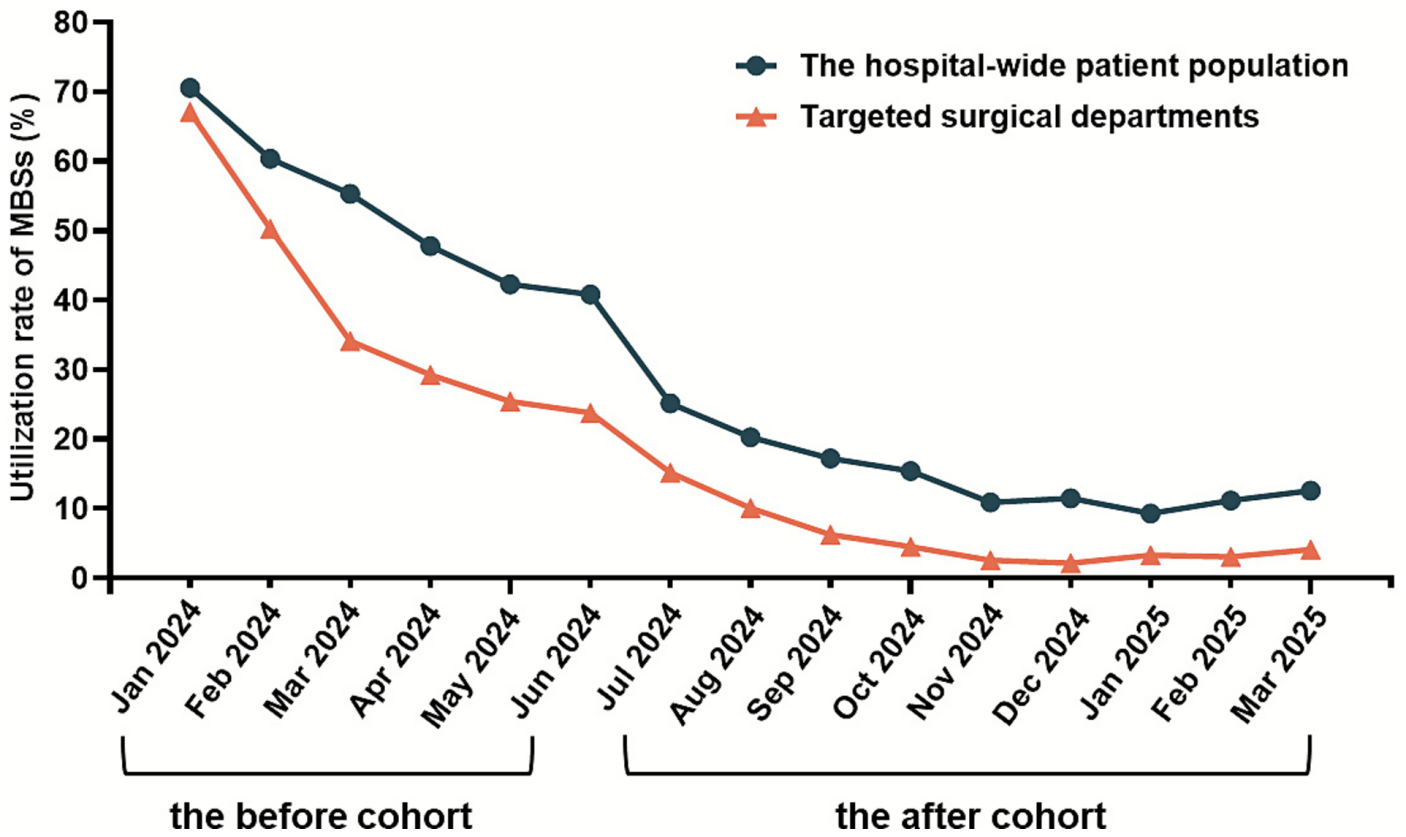
Changes in utilization rate of MBSs before and after the formulary prescription-based nutrition therapy protocol establishment.

Following the protocol implementation, statistically significant reductions in the MBSs utilization rates were observed in both targeted surgical departments and the hospital-wide patient population (*p* < 0.01, Table 2). The MBSs usage rates decreased from peak values of 67.09 to 4.07% in the targeted surgical departments, and from 70.56 to 12.55% among the hospital-wide inpatients. Standardization of PN administration practices showed progressive improvement, as illustrated in Figure 2.

**Table 2:** Utilization rate of MBSs before and after the formulary prescription-based nutrition therapy protocol establishment [case^a^ (%)].

Departments	The before cohort (January to June 2024)			The after cohort (July 2024 to March 2025)			*P-*value
	PN administrations	MBSs	AIOs	PN administrations	MBS	AIOs	
Targeted surgical departments							
General surgery	1,685	632 (37.5)	1,053 (62.5)	2,199	106 (4.8)	2093 (95.2)	<0.01
Thoracic surgery	202	90 (44.4)	112 (55.6)	344	37 (10.7)	307 (89.3)	<0.01
Total	1887	722 (38.3)	1,165 (61.7)	2,543	143 (5.6)	2,400 (94.4)	<0.01
The hospital-wide patient population	3,557	1878 (52.8)	1,679 (47.2)	4,281	634 (14.8)	3,647 (85.2)	<0.01

PN, parenteral nutrition; MBSs, multiple bottle systems; AIOs, all-in-one admixtures. ^a^ Number of PN administrations.

### 3.2 Formulary prescription-based nutrition therapy enhanced the personalization of PN regimens

**Figure 3:**
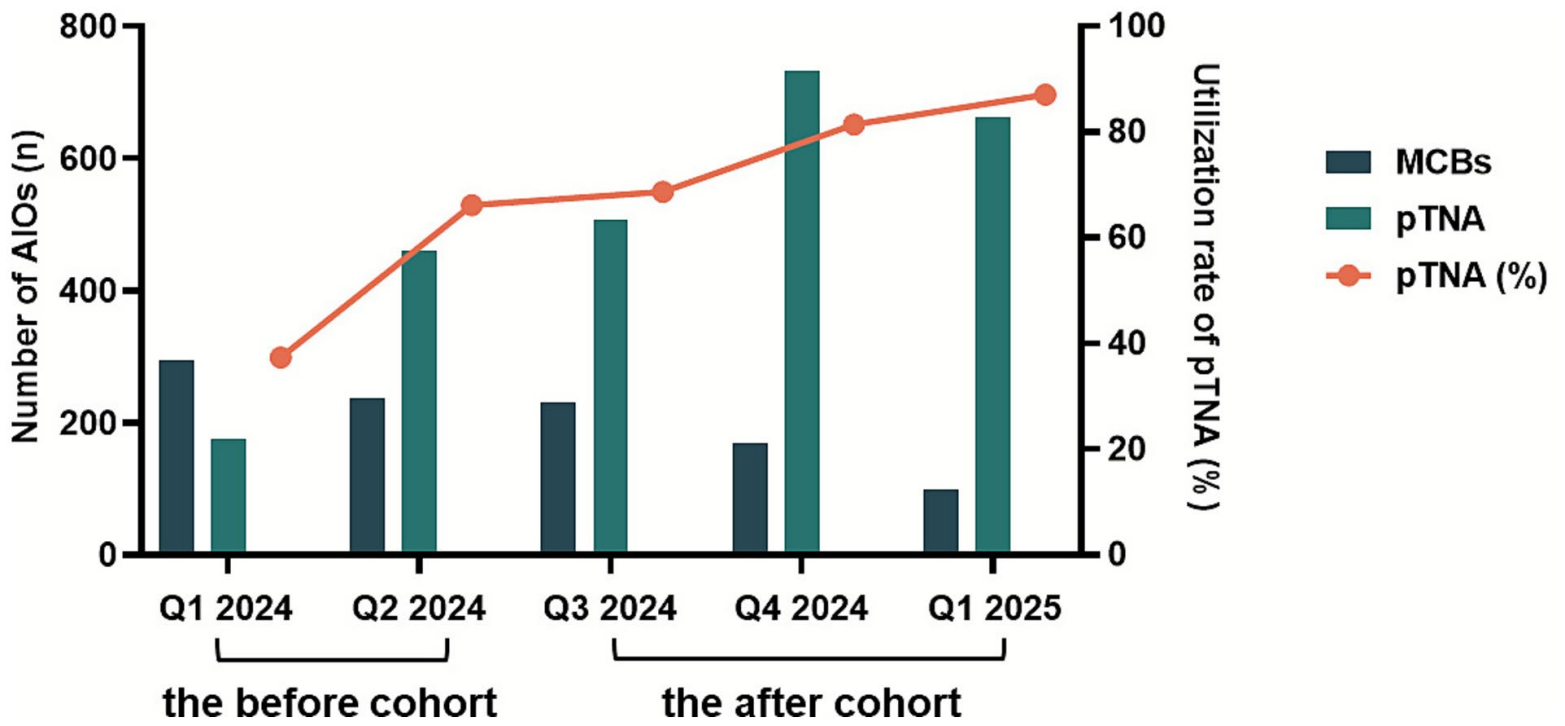
Changes in utilization rate of pTNA before and after the formulary prescription-based nutrition therapy protocol establishment.

Following the protocol implementation, the utilization rate of pTNA among all-in-one PN prescriptions significantly increased from 54.5 to 79.3% in the targeted surgical departments (*p* < 0.01, Table 3). As presented in Figure 3, the utilization rate of pTNA changed significantly over the study period. Specifically, approximately 65% of pTNA prescriptions were derived from PN formulary prescriptions embedded within the HIS.

**Table 3:** Utilization rate of pTNA before and after the formulary prescription-based nutrition therapy protocol establishment [case^a^ (%)].

Targeted surgical departments	The before cohort (January to June 2024)			The after cohort (July 2024 to March 2025)			*P* value
	AIOs	MCBs	pTNA	AIOs	MCBs	pTNA	
General surgery	1,053	440 (41.8)	613 (58.2)	2093	348 (16.6)	1745 (83.4)	<0.01
Thoracic surgery	112	90 (80.4)	22 (19.6)	307	150 (48.9)	157 (51.1)	<0.01
Total	1,165	530 (45.5)	635 (54.5)	2,400	498 (20.8)	1902 (79.3)	<0.01

AIOs, all-in-one admixtures; MCBs, multi-chamber bags; pTNA, personalized total nutrient admixture. ^a^ Number of PN administrations.

### 3.3 Formulary prescription-based nutrition therapy optimized the approaches of perioperative nutrition support

**Figure 4:**
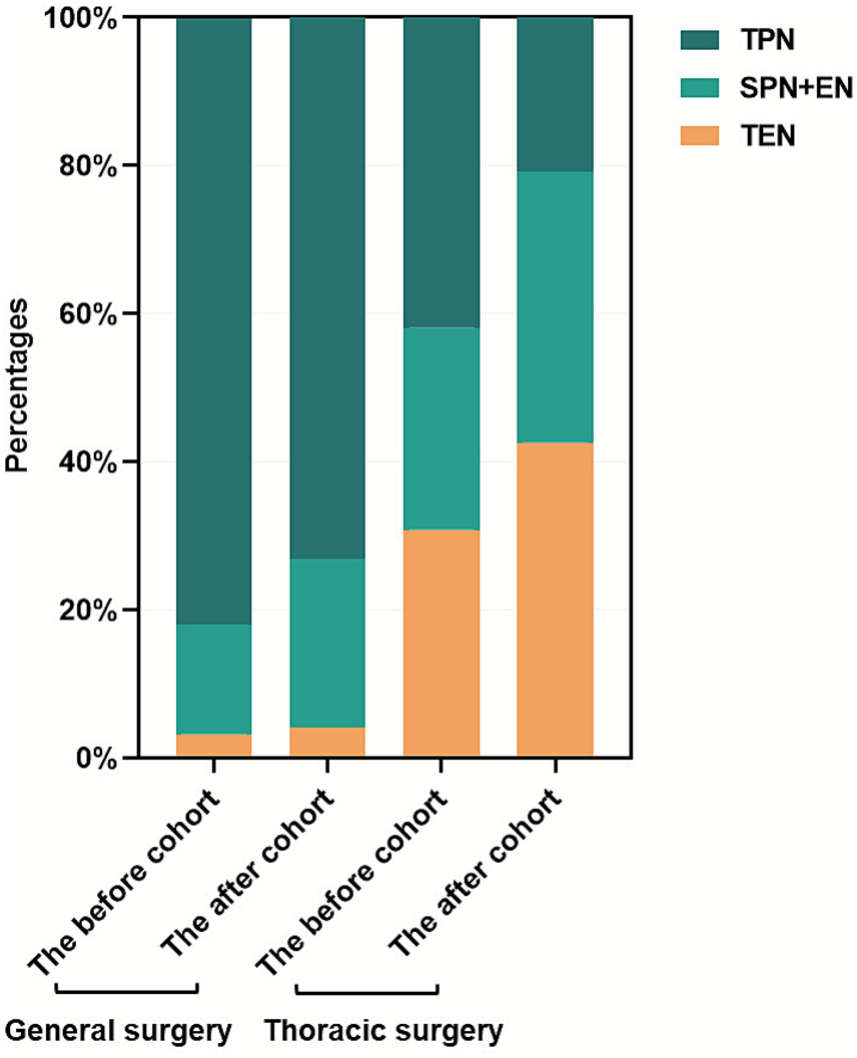
Changes in nutrition therapy approaches in targeted surgical departments before and after the formulary prescription-based nutrition therapy protocol establishment.

Following the protocol implementation, significant changes in nutrition therapy approaches were observed in the targeted surgical departments (Table 4 and Figure 4). A marked reduction in TPN therapy (60.9% vs. 52.9%) was observed concurrently with increased adoption of combined SPN and EN therapy (21.4% vs. 28.1%). Notably, the thoracic surgery department demonstrated a particularly significant transition toward TEN utilization (30.8% vs. 42.6%).

**Table 4:** Changes in nutrition therapy approaches in targeted surgical departments before and after the formulary prescription-based nutrition therapy protocol establishment [case (%)].

Targeted surgical departments	Nutrition therapy approaches^a^	The before cohort (January to June 2024) (*n*, %)	The after cohort (July 2024 to March 2025) (*n*, %)	*p-*value
General surgery	TEN	5 (3.2)	17 (4.1)	0.642
	SPN + EN	23 (14.8)	95 (22.7)	0.038
	TPN	127 (81.9)	306 (73.2)	0.031
Thoracic surgery	TEN	53 (30.8)	113 (42.6)	0.013
	SPN + EN	47 (27.3)	97 (36.6)	0.044
	TPN	72 (41.9)	55 (20.8)	<0.01
Total	TEN	58 (17.7)	130 (19.0)	0.620
	SPN + EN	70 (21.4)	192 (28.1)	0.023
	TPN	199 (60.9)	361 (52.9)	0.017

TNE, total enteral nutrition; EN, enteral nutrition; SPN, supplemental parenteral nutrition; TPN, total parenteral nutrition. ^a^ Nutrition therapy approaches excluded oral nutritional supplements (ONS) or multiple bottle systems (MBSs).

### 3.4 Formulary prescription-based nutrition therapy improved the postoperative nutrition status in patients with GI cancers

**Figure 5:**
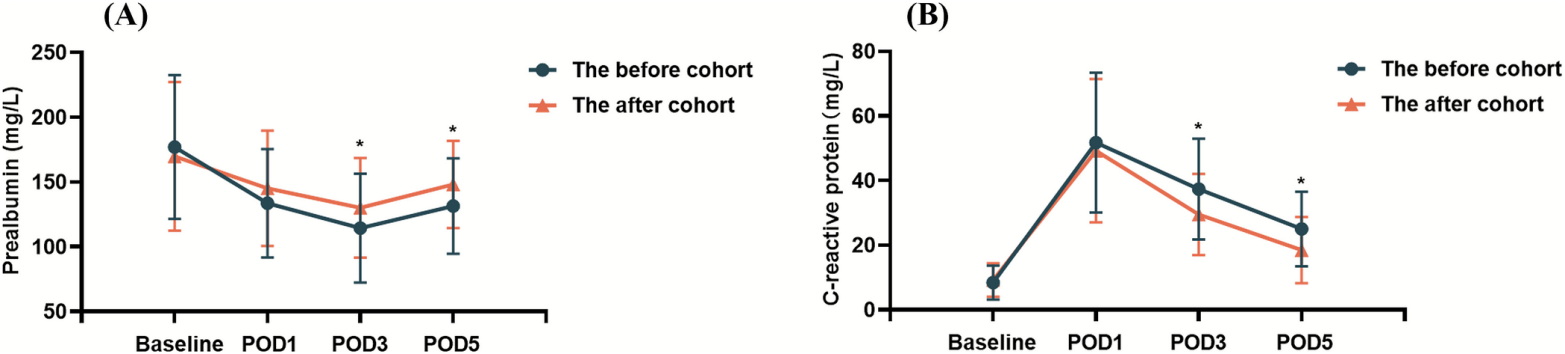
Changes in the postoperative **(A)** prealbumin and **(B)** C-reactive protein levels among patients with GI cancers. *p < 0.05.

As shown in Table 5 and Figure 5, compared with preoperative level, postoperative decline of albumin and prealbumin levels occurred in both the before cohort and the after cohort. However, the after cohort demonstrated significantly higher prealbumin levels on both postoperative day 3 (POD 3) and postoperative day 5 (POD 5) compared with the before cohort (114.40 ± 41.95 vs. 130.09 ± 38.55 mg/L on POD 3 and 131.45 ± 36.88 vs. 148.02 ± 33.69 mg/L on POD 5, *p* < 0.05). The C-reactive protein levels in the after cohort were significantly lower than those in the before cohort on both POD 3 and POD 5 (37.34 ± 15.65 vs. 29.50 ± 12.57 mg/L on POD 3 and 25.05 ± 11.54 vs. 18.53 ± 10.31 mg/L on POD 5, *p* < 0.05). No significant difference was found in the albumin levels between the two cohorts on POD3 and POD5.

**Table 5:** Changes in the postoperative albumin, prealbumin and C-reactive protein levels among patients with GI cancers^a^.

Indicators	The before cohort (January to June 2024, *n* = 52)	The after cohort (July 2024 to March 2025, *n* = 87)	*p-*value
Albumin (g/L)			
Baseline	39.62 (4.32)	38.84 (4.43)	0.322
POD1	34.14 (4.08)	35.53 (4.51)	0.070
POD3	37.16 (3.17)	38.43 (3.88)	0.089
POD5	38.45 (4.97)	38.97 (4.09)	0.518
Prealbumin (mg/L)			
Baseline	178.31 (56.88)	169.69 (57.37)	0.398
POD1	133.57 (41.82)	145.15 (44.64)	0.132
POD3	114.40 (41.95)	130.09 (38.55)	0.034
POD5	131.45 (36.88)	148.02 (33.69)	0.030
C-reactive protein (mg/L)			
Baseline	8.44 (5.28)	9.21 (5.22)	0.617
POD1	51.75 (21.60)	49.26 (22.21)	0.577
POD3	37.34 (15.65)	29.50 (12.57)	0.027
POD5	25.05 (11.54)	18.53 (10.31)	0.021

GI, gastrointestinal; POD, postoperative day. ^a^ Data were presented as mean (SD) unless otherwise indicated.

### 3.5 Formulary prescription-based nutrition therapy accelerated the postoperative recovery of patients with GI cancers

The after cohort exhibited significantly shorter duration of postoperative PN therapy (8.2 ± 3.3 vs. 6.3 ± 2.6 days) and length of postoperative hospital stay (14.9 ± 5.1 vs. 12.2 ± 4.2 days) (*p* < 0.05), along with earlier recovery of bowel function (3.7 ± 0.5 vs. 2.3 ± 0.4 days) compared with the before cohort, as detailed in Table 6. No significant differences were found in the rest of the recovery indicators between the two cohorts.

**Table 6:** Changes in the postoperative recovery of patients with GI cancers^a^.

Indicators	The before cohort (January to June 2024, *n* = 52)	The after cohort (July 2024 to March 2025, *n* = 87)	*p-*value
Duration of postoperative PN therapy (days)	8.2 (3.3)	6.3 (2.6)	<0.01
Anal exhaust time (days)	3.7 (0.5)	2.3 (0.4)	0.025
Duration of abdominal drainage (days)	9.6 (2.2)	9.4 (1.8)	0.342
Length of postoperative hospital stay (days)	14.9 (5.1)	12.2 (4.2)	0.021
Length of total hospital stay (days)	18.6 (5.4)	16.9 (5.2)	0.095

GI, gastrointestinal; PN, parenteral nutrition. ^a^ Data were presented as mean (SD) unless otherwise indicated.

### 3.6 Formulary prescription-based nutrition therapy reduced postoperative complications in patients with GI cancers

The incidence of postoperative complications was systematically compared between the two cohorts. Overall, patients in the after cohort had slightly fewer complications during the monitoring period, but these differences were not significant, as shown in Table 7.

**Table 7:** Changes in the postoperative complications in patients with GI cancers^a^.

Complications	The before cohort (January to June 2024, *n* = 52)	The after cohort (July 2024 to March 2025, *n* = 87)	*p-*value
GI intolerance complications	32 (61.5)	49 (56.3)	0.546
PN-related complications	5 (9.6)	5 (5.7)	0.393
Infectious complications	7 (13.5)	8 (9.2)	0.433
Gastroparesis	2 (3.8)	3 (3.4)	0.903
Intestinal obstruction	1 (1.9)	NA	/
Anastomotic leak	2 (3.8)	2 (2.3)	0.598

GI, gastrointestinal; PN, parenteral nutrition; NA, not applicable. ^a^ Data were presented as number (percentage) of participants unless otherwise noted.

## 4 Discussion

This current study demonstrated that a formulary prescription-based nutrition therapy can systematically address the challenges associated with the utilization of PN drugs. PN administration methods were standardized, individualized PN regimens were enhanced, perioperative nutrition therapy approaches were optimized in the surgical patient population. Additionally, the pharmacist-led PN standardization improved the postoperative clinical outcomes in patients with GI cancers.

The administration of PN has become more standardized. In the before cohort, glucose, amino acids, and lipid emulsions were often administered as separate infusions, a method referred to as MBSs. This approach has several limitations, including a high risk of infection, an increased incidence of phlebitis and metabolic complications, prolonged infusion times, and an increased nursing workload (15). Compared to MBSs, all-in-one PN can enhance the nutrient utilization efficiency, minimize metabolic complications, reduce infection risk, and better align with the body’s physiological metabolic processes (14, 18). It is therefore the recommended mode of PN administration. During the study period (January 2024 to March 2025), the utilization rate of MBSs showed an overall downward trend both hospital-wide and in targeted surgical departments. In the before cohort, the decline in the use of MBSs was primarily attributed to the pre-prescription review system intercepting orders for this infusion method. However, this also created another issue: extraordinary PN prescriptions prescribed by clinicians, especially those with incompatibility issues and inappropriate nutrients’ ratios were frequently intercepted by the computerized PN management system in PIVAS. This led to reluctance among physicians to prescribe all-in-one PN orders. The implementation of PN formulary prescription effectively resolved this conflict and was pivotal in achieving and maintaining a sustained reduction in the utilization rate of MBSs to a lower level in the after cohort. Concurrently, it significantly enhanced the workflow efficiency for both physicians and prescription-reviewing pharmacists.

The clinical guidelines of the ASPEN suggested that commercially available MCBs, together with hospital-customized personalized TNA, can optimally meet the needs of the majority of patients (13). Some studies have indicated that MCBs with nutrients mixed and activated by squeezing when in use significantly enhanced the efficiency and safety of PN solutions and reduced medical costs (19, 20). While MCBs simplify the compounding process, their nutrient composition and solution volume are relatively fixed. To ensure the stability of PN, only limited supplemental additions of vitamins and electrolytes strictly following the manufacturers’ provided stability data can be made to MCBs (21). However, in our clinical practice of perioperative PN therapy for surgical patients, we observed significant variations in individual requirements for both fluid volume and energy content among different patients. Specifically, MCBs often provided excessive fluid volume for certain populations, particularly elderly patients with impaired cardiac function or those requiring supplemental PN therapy only. Conversely, switching to lower-volume MCBs resulted in high osmolality for patients without central venous access. Therefore, we developed and successfully implemented a series of standardized PN formulary prescriptions with graded volumes (from low to high) and progressively increasing energy and protein content. These PN formulary prescriptions effectively addressed the limitations of MCBs. Furthermore, as these patients were clinically stable and did not require specialized nutrition pharmacist consultations for customized PN regimen design, this approach significantly conserved medical resources and optimized the nutrition therapy pathway for perioperative patients. Notably, our hospital is a large comprehensive public hospital, equipped with advanced sterile compounding equipment, standardized compounding procedures for PN and highly skilled medical staff, capable of preventing errors during the compounding process.

The formulary prescription-based perioperative nutrition medication pathways have fundamentally altered nutrition therapy approaches in surgical care. The ESPEN and the Enhanced Recovery After Surgery (ERAS) Society guidelines (12, 22) recommend that EN should be implemented for patients after surgery as soon as possible if the GI tract works. However, in many cases, energy delivery in postsurgical patients using EN alone is less than the estimated requirements for various reasons. A randomized clinical trial (23) and previous studies (24, 25) have demonstrated that SPN combined with EN can substantially improve energy delivery after surgery and prevent energy deficits during the initial postoperative days. Moreover, SPN combined with EN was associated with reduced nosocomial infections in patients undergoing surgery and seems to be a favorable strategy for patients with high nutritional risk and poor tolerance to EN after major surgery. In the after cohort in this study, both targeted surgical departments showed a reduction in TPN usage and an increase in the approach of SPN combined with EN. The utilization rate of TEN increased in patients undergoing pulmonary and esophageal surgeries in the thoracic surgery department, as these procedures result in minimal disruption to postoperative GI function.

To investigate whether the formulary prescription-based nutrition therapy protocol directly affected the recovery of patients’ nutrition status, this study continued to analyze the changes in the expression level of negative (albumin and prealbumin) and positive (C-reactive protein) acute-phase proteins in patients with GI cancers. Serum albumin is a recognized factor that reflects the nutrition status of patients and has been widely used as a predictor of outcome (26, 27), including postoperative outcome of cancer patients (28). Prealbumin is another commonly used indicator of nutrition status. Low prealbumin levels indicate malnutrition, and are associated with sarcopenia and adverse prognosis (29). Due to its short half-life, prealbumin is more sensitive than albumin in faster reflecting the function of liver synthesis and secretion of protein, and the acute changes in nutrition status in the short-term (30). In some cases, prealbumin levels have been found to be the best independent nutritional predictor (31). Serum C-reactive protein significantly increased after 6–8 h of inflammation, which can be used as an early diagnostic marker of surgical stress (32). C-reactive protein has also been proved to be an effective predictor of postoperative complications (33). In clinical practice, prealbumin and C-reactive protein are often measured simultaneously. Their opposing directional changes form a “scissor-shaped” divergence, providing a more comprehensive assessment of inflammatory status and nutritional conditions. The present study showed that prealbumin and C-reactive protein were significantly lower or higher, respectively, on POD 1 and POD 3 for the two cohorts when compared with the preoperative period. Nevertheless, following the postoperative nutrition therapy, the after cohort demonstrated higher prealbumin levels and lower C-reactive protein levels on both POD3 and POD5 compared with the before cohort. The postoperative serum prealbumin and C-reactive protein levels were all improved in the after cohort, suggesting that the formulary prescription-based nutrition therapy protocol improved the postoperative nutrition status and attenuated the body’s response to the stress of surgical trauma in patients with GI cancers.

The nutrition status of patients is associated with the postoperative recovery and the incidence of postoperative complications. Our results revealed that the duration of postoperative PN therapy, the postoperative GI function recovery time and the length of postoperative hospital stay were significantly shorted in the after cohort. These indicated that formulary prescription-based nutrition therapy in patients with GI cancers allowed for a rapid rehabilitation. However, there were no statistical difference between the two cohorts in terms of the incidence of complications, which was probably due to the small sample size and the short follow-up periods.

There were some certain limitations to this study. First, as a retrospective study rather than a prospective, randomized, controlled study, the results may be influenced by several interfering factors. Second, the nutrition therapy strategies investigated were predominantly focused on surgical departments, and the clinical outcome measures were confined to patients undergoing GI cancer surgery. Several indicators of postoperative outcome might be affected by medical policy or clinical practice. For example, the discharge time of patients may be affected by the lack of beds and policies related to the rate of bed turnover. Third, the absence of follow-up data in this study prevented an assessment of long-term clinical outcomes of patients, including prognosis and complications. Notably, with the continuous advancement and refinement of the National Centralized Drug Procurement (NCDP) policy (34, 35), the variety of parenteral and enteral nutrition medicines available in our hospital have been subsequently adjusted, accompanied by changes in their prices. In light of this, the present study did not analyze the nutrition therapy costs for patients. Therefore, future work should focus on (1) implementing and refining the precise management system for clinical nutrition therapy, and expanding the coverage to include additional targeted departments and patient populations, (2) conducting multicenter cluster randomized controlled trials and economic evaluation, in order to further reflect the impact of our nutrition interventions on patients’ clinical efficacy and medical costs.

## 5 Conclusion

In summary, the formulary prescription-based nutrition therapy protocol which guided by the pharmacists, significantly promoted the rationality of the use of PN agents, the individualization of perioperative nutrition therapy, and the standardization of perioperative nutrition therapy pathway. In addition, it is also beneficial to improve the short-term postoperative nutritional indicators and recovery outcomes of patients. Subsequent work will center on evaluating the clinical efficacy and cost-effectiveness of these nutrition therapy management protocols through multicenter, prospective clinical trials.

## Data Availability

The original contributions presented in the study are included in the article/supplementary material, further inquiries can be directed to the corresponding author/s.
